# Supplementary material for: Withaferin a Attenuates Retinal Ischemia-Reperfusion Injury via Akt-Dependent Inhibition of Oxidative Stress
Source: Cells. 2022 Oct 2;11(19):3113. doi: 10.3390/cells11193113 (PMC9563317; doi:10.3390/cells11193113)
Supplement: Supplementary file 1 [file cells-11-03113-s001.zip › cells-1914543-supplementary.pdf]

Table S1: Real-time PCR primers

Genes	Forward Primer (5'-3')	Reverse Primer (5'-3')
HMOX1	AAGACTGCGTTCCTGCTCAAC	AAAGCCCTACAGCAACTGTCG
HSPA1A	CGGGGTACCGCCTTTCAGGTTCACAATC	CCCAAGCTTCAATCAGCCGCTTCG
HSPA1B	TTTGAGGGCATCGACTTCTACA	CCAGGACCAGGTCGTGAATC
OSGIN1	CCCGGTCATCATTGTGGGTAA	GCTTCGTGTAGGGTGTGTAGC
DNAJB1	AAGGCATGGACATTGATGACC	GGCCAAAGTTCACGTTGGT
CLCF1	TTTCAACGAGCCAGACTTCAAC	GAGGCCACGCAAGTAACACA
DNAJA4	GGGATGTTTATGACCAAGGCG	GCCAATTTCTTCGTGACTCCA
ADAMTS4	GAGGAGGAGATCGTGTTTCCA	CCAGCTCTAGTAGCAGCGTC
PRDX1	CCACGGAGATCATTGCTTTCA	AGGTGTATTGACCCATGCTAGAT
TXNRD1	ATATGGCAAGAAGGTGATGGTCC	GGGCTTGTCCTAACAAAGCTG
IFNE	GGCCTCTACCACTATCTTCTCTC	ACACTGCTGAATTGACAAGGTTT
LDLR	TCTGCAACATGGCTAGAGACT	TCCAAGCATTCGTTGGTCCC
Actb (β- actin)	CATGTACGTTGCTATCCAGGC	CTCCTTAATGTCACGCACGAT
